# Prediction models for basal endogenous losses of crude protein and amino acids in pigs

**DOI:** 10.5713/ab.24.0197

**Published:** 2024-06-25

**Authors:** Noa Park, Hansol Kim, Beob Gyun Kim

**Affiliations:** 1 Department of Animal Science, Konkuk University, Seoul 05029, Korea

**Keywords:** Amino Acids, Basal Endogenous Losses, Crude Protein, Prediction Equation, Swine

## Abstract

**Objective:**

The objectives were to validate a previously published equation for estimating basal endogenous losses (BEL) of crude protein (CP) in pigs fed nitrogen-free diets and to develop prediction equations for BEL of CP and amino acids (AA).

**Methods:**

A total of 139 observations from 123 experiments in 117 papers that determined the BEL of CP and AA in pigs were collected. For the validation of the previous equation for the BEL of CP, 94 observations that were not used for developing the previous equation were used. All observations were used to develop novel equations for estimating BEL of CP in pigs based on the initial body weight (IBW).

**Results:**

The validation study indicated that the slope for BEL of CP, representing a linear bias, was less than zero (−0.56; standard error [SE] = 0.130; p<0.001). The intercept for BEL of CP, representing a mean bias, was less than zero (−3.21; SE = 0.488; p<0.001). The models for estimating BEL of CP (g/kg dry matter intake) in pigs fed a nitrogen-free diet were developed: 20.36–0.077×IBW with R^2^ = 0.11 and p<0.001 and 20.80×e^(−0.00475×IBW)^ with R^2^ = 0.12 and p<0.001. Novel linear models for estimating BEL of AA were developed using BEL of CP as the independent variable.

**Conclusion:**

The accuracy of the previous equation for estimating BEL of CP in pigs has been improved by reflecting additional data from recent publications. In the novel linear models for estimating BEL of AA of pigs, BEL of CP was used as an independent variable.

## INTRODUCTION

One of the main goals in swine production is efficient protein deposition in pigs with the least-cost formulation. For efficient protein deposition with a cost-effective formulation in pigs, accurate protein and amino acids (AA) requirements for pigs, as well as the AA profile of feed ingredients, should be available [1]. Thus, experiments determining digestible AA in feed ingredients have been extensively reported [2–5]. When expressing digestible crude protein (CP) and AA in feed ingredients, standardized ileal digestible CP and AA are more widely used rather than apparent ileal digestible nutrient concentrations due to the additivity in a mixed diet [6]. To calculate standardized ileal digestibility (SID) values, both the apparent ileal digestibility of CP and AA and basal endogenous losses (BEL) of CP and AA, often expressed as gram per kilogram dry matter intake (DMI), are needed [6].

Accurate BEL of CP and AA are critical for determining accurate SID of CP and AA in feed ingredients. To determine BEL of CP and AA, researchers employ nitrogen (N)-free diets [2], casein diets [7], or regression methods [8]. If the determination of BEL of CP and AA is challenging due to certain circumstances, researchers can use published values of BEL of CP and AA [9], averaged values of BEL of CP and AA [10], or prediction equations [11]. Among these methods, the use of prediction equations for estimating BEL of CP and AA would be preferred rather than using averaged or published values as the equations consider physiological characteristics of pigs such as body weight (BW) and feed intake. Park et al [11] reported prediction equations for estimating BEL of CP and indispensable AA based on factors that affect BEL of CP and indispensable AA, which are initial BW (IBW) and feed intake-to-maintenance feed intake ratio (FI:MFI) using data obtained from pigs fed N-free diets. However, the equations published by Park et al [11] have limitations, including the absence of equations for estimating BEL of dispensable AA and the use of a relatively small dataset (n = 30 to 42) for the equation development. In the current study, to overcome these limitations, the previous prediction equation [11] for estimating BEL of CP (g/kg DMI) in pigs fed N-free diets was validated using more recent data. Additionally, novel prediction equations for estimating BEL of CP and AA were developed using a larger size dataset.

## MATERIALS AND METHODS

### Data collection and investigation

A dataset was created using online database of Google Scholar based on the following keywords: amino acids, basal endogenous losses, crude protein, pigs, and standardized ileal digestibility. The studies found through the literature search were then manually screened based on the title and experimental information. The analyzed composition of N-free diets, IBW of pigs, feeding level of pigs, and BEL of CP and AA (g/kg DMI) of pigs were collected. All data were from the pigs fed N-free diets. A total of 155 observations from 128 experiments in 137 literature that determined the BEL of CP and AA of pigs were collected. These research articles were published in 1991 to 2023. Maintenance feed intake of each experiment was calculated by dividing metabolizable energy for maintenance (i.e., 197×IBW^0.60^; NRC [12]) by the metabolizable energy concentration of the N-free diet [11]. Then, the FI:MFI indicating the number of times the maintenance feed intake was calculated based on the feed intake data and the metabolizable energy concentration of the experimental diet unless the FI:MFI is provided in the paper. However, if the FI:MFI exceeded the value calculated using the feed intake and metabolizable energy data suggested in the NRC [12], feed intake of the pigs was assumed to be the same as the amount suggested in the NRC [12]. This assumption was necessary as the high FI:MFI values were not practical considering general feed intake of crossbred pigs. For instance, although the FI:MFI was 4.9 in Otto et al [13], the value of 3.0 FI:MFI was used based on the NRC [12].

### Statistical analysis

Before the statistical analysis of the obtained dataset, outliers were removed from the dataset for the final statistical analysis. Sixteen observations with Cook’s distance values greater than Fox’s criterion (0.026) were excluded resulting in 139 observations left [14]. The mean, standard deviation, and coefficient of variation (CV) for all variables were calculated. The data were analyzed using the CORR procedure of SAS (SAS Inst. Inc., Cary, NC, USA) to determine correlation coefficients among variables. The accuracy of the previously published equation for BEL of CP was tested using regression analysis. The regression analysis was conducted using the REG procedure of SAS, with measured minus predicted BEL of CP as a dependent variable and the predicted BEL of CP minus the mean predicted BEL of CP as an independent variable [15]. In the linear regression, the intercept and the slope represented a mean bias and a linear bias, respectively. The results were presented with statistical parameters of standard error (SE) and p-value. The 94 observations which were not included in the dataset for generating equations for BEL of CP in the previous study [11] were used to validate the previously published equation. A break point of BW of pigs for BEL of CP was estimated by a one-slope broken-line model using the NLIN procedure of SAS [16]. The prediction equation for BEL of CP was developed using the REG and NLIN procedures of SAS with IBW, FI:MFI, or both included as independent variables. The prediction equations for BEL of AA were developed using the REG procedure of SAS with BEL of CP as an independent variable. The statistical significance was declared at an alpha level less than 0.05.

## RESULTS

The BEL of CP averaged 17.1 g/kg DMI with a range of 6.1 to 29.1 g/kg DMI (Table 1). Among the BEL of AA, the BEL of Pro showed the greatest mean value of 4.542 g/kg DMI followed by BEL of Gly (1.536 g/kg DMI). The BEL of Ser had the largest variability (CV = 78.2%) followed by BEL of Pro (CV = 65.0%). The IBW of pigs was positively correlated (r = 0.40; p<0.001) with FI:MFI, but negatively correlated with BEL of CP (r = −0.34; p<0.001) and BEL of all indispensable AA (r<−0.17; p<0.05; Table 2). The FI:MFI was negatively correlated with BEL of CP (r = −0.20; p<0.05) and most indispensable AA (r<−0.20; p<0.05). The BEL of CP was positively correlated with all indispensable AA (r> 0.25; p<0.01).

**Figure 1:**
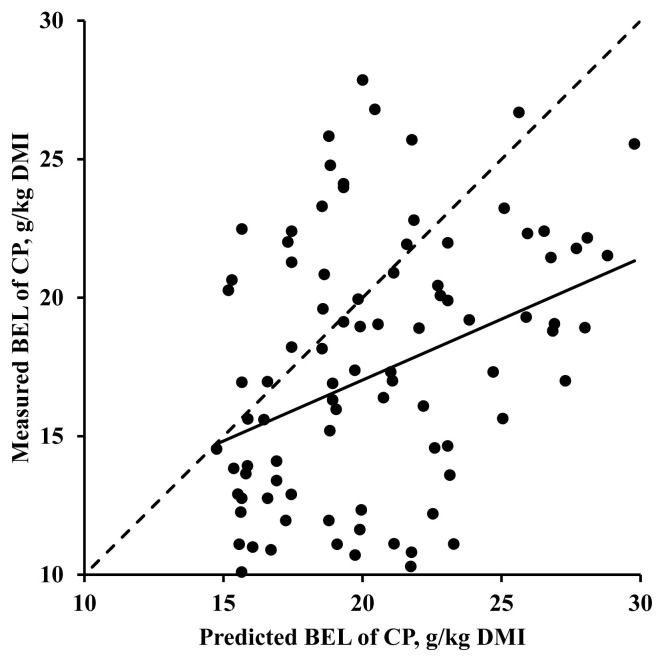
Validation of the prediction equation suggested by Park et al [11] for basal endogenous losses (BEL) of crude protein (CP) based on initial body weight and feed intake-to-maintenance feed intake ratio. A total of 94 data from 85 studies published between 1991 and 2023 were used. The method for estimating BEL of CP (g/kg dry matter intake; DMI) was nitrogen-free diets. Based on the regression analysis of measured minus predicted BEL of CP on the BEL of CP adjusted to the mean as zero, the slope (−0.56±0.130; p<0.001) and the intercept (−3.21±0.488; p<0.001) were less than zero.

**Figure 2:**
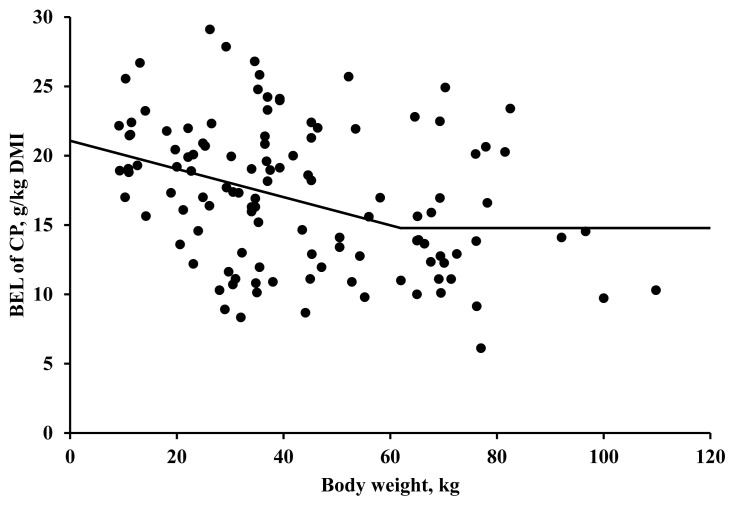
A one-slope broken-line analysis of the basal endogenous losses (BEL) of crude protein (CP) according to body weight of pigs (n = 117). The break point of BEL of CP (g/kg dry matter intake; DMI) was estimated based on following equation: BEL of CP (g/kg DMI) = 14.78+0.102×(62.0–body weight) where body weight less than 62.0 (R^2^ = 0.12, root mean square error = 4.89, and p<0.001).

The validation study indicated that the slope for BEL of CP (Figure 1), representing a linear bias, was less than zero (−0.56; SE = 0.130; p<0.001). The intercept for BEL of CP, representing a mean bias, was also less than zero (−3.21; SE = 0.488; p<0.001). Based on the one-slope broken-line analysis (Figure 2), the BEL of CP (g/kg DMI) in pigs decreased as BW increased for pigs less than 62.0 kg, but constant when the BW of pigs was greater than 62.0 kg (R^2^ = 0.12, root mean square error = 4.89, and p<0.001).

The best-fit linear model for BEL of CP (g/kg DMI) was: 20.36–0.077×IBW with root mean square error = 4.87, r^2^ = 0.11, and p<0.001 (Table 3). The best-fit nonlinear model for BEL of CP (g/kg DMI) was: 20.80×e^(−0.00475×IBW)^ with root mean square error = 4.86, r^2^ = 0.12, and p<0.001. Prediction equations for BEL of AA (g/kg DMI) were developed: BEL of Lys = 0.017×BEL of CP+0.148 with r^2^ = 0.28 and p<0.001; and BEL of Thr = 0.020×BEL of CP+0.217 with r^2^ = 0.43 and p<0.001 (Table 4).

## DISCUSSION

Due to the variability among laboratories and within laboratories of BEL of AA, the BEL of AA have been suggested to be measured in each individual experiment [1]. However, the prediction equations for BEL of AA can be used as an alternative method for the situations when the BEL of AA cannot be measured. Thus, the equations for estimating BEL of AA developed by Park et al [11] would be useful. However, the equations by Park et al [11] did not include dispensable AA and the data size for the modeling was relatively small. In the present work, thus, the previously published prediction equations were validated using recent data and novel prediction equations for estimating BEL of dispensable AA in addition to indispensable AA in pigs were developed based on a relatively large dataset. The models for BEL of dispensable AA become more important as dispensable AA are potentially limiting in low-CP diets that are often used for swine diets.

The BEL of CP and AA have been measured by various methods such as N-free diets [2,17], casein diets [7], and regression methods [8]. In the present study, the data for BEL of AA measured using N-free diet were used as the N-free diet method has been most widely used in pigs. In addition, the BEL of CP and AA are affected potentially by methods used [18].

The mean value for BEL of CP (17.1 g/kg DMI) in the present study is fairly comparable to the value of 17.3 g/kg DMI reported by Adeola et al [1], but is slightly less than the value of 20.2 g/kg DMI reported by Lee and Stein [19] who collected data from the University of Illinois during 2010 to 2020. It is speculated that the BEL of CP measured at the University of Illinois were a bit greater than those measured in other institutes potentially due to the less IBW in the data by Lee and Stein [19]. In the present work, the negative correlation between IBW and the BEL of CP was observed for the IBW less than 62 kg (Figure 2).

The BEL of Pro was the greatest among the AA with great variability, which is consistent with previous studies [18,19]. This observation may be associated with the mobilization of body protein under negative N balance when pigs fed the N-free diet [1]. In addition, de Lange et al [20] observed that BEL of Pro in pigs fed the N-free diet with intravenous injection of a balanced AA mixture was reduced compared with the administration of saline for pigs, suggesting that mobilized muscle under abnormal status in pigs can be transformed to Gly and Pro in the intestinal tissue after metabolism.

The specific reason for the overestimation of BEL of CP in the previous equation [11] remains unclear. However, the effects of adaptation period may have affected the BEL of CP [17,21]. In the study by Park et al [11], the average adaptation period prior to ileal collection in the dataset was approximately 5.4 days which was longer than the adaptation period in the dataset used in the present study. Moreover, Park et al [11] used some data with a FI:MFI greater than 3.0 for developing equations without correction. These may have affected the accuracy of the previous models. The slope bias in the validation test indicates that the previous equation overestimates the BEL of CP even more for greater values of BEL of CP.

In the present study, IBW and feeding levels (i.e., FI:MFI) were considered important contributors affecting the BEL of CP. In agreement, Park et al [11] used the IBW and FI:MFI of pigs as independent variables for estimating the BEL of CP and AA. The influence of IBW and feeding levels on the BEL of CP has been reported in animal experiments. The effect of BW on BEL of CP was demonstrated by Hess and Sève [22], who observed a 45% decrease in BEL of N (g/kg DMI) in 77-kg pigs compared with 45-kg pigs fed an N-free diet. Similarly, Pahm et al [23] also observed the greater BEL of CP and AA (g/kg DMI) in 37-kg pigs compared with 76-kg pigs fed an N-free diet. The influence of BW on the BEL of CP and AA (g/kg DMI) was also observed in broiler chicks by Barua et al [24], who suggested that the greater BEL of CP and AA (g/kg DMI) in younger broilers would be due to the immaturity of the gastrointestinal tract.

Regarding the influence of feeding level on the BEL of CP (g/d) in pigs, Moter and Stein [25] reported that the BEL of CP linearly increased with an increase in the FI:MFI at 1 to 3 in 70.3-kg pigs. Additionally, Hess and Sève [22] also reported that increasing feed intake linearly increased the BEL of CP (g/kg DMI). The reason is that as feed intake increases, digestive enzyme secretion increases [22,25], leading to a linear increase in the BEL of CP (g/d) despite the constant amount of inevitable gut BEL of CP (g/d) that is secreted regardless of the feed intake [25]. However, previous studies [22,25] also showed that the BEL of CP expressed as gram per kilogram DMI decreased when pigs consumed a greater quantity of N-free diet. The potential reason is that the amount of inevitable gut BEL of CP relative to feed intake is constant regardless of feed intake [25]. Thus, when dividing BEL of CP (g/d) by DMI (kg/d), a decreasing BEL of CP (g/kg DMI) is observed when pigs are fed a greater feed intake. However, Stein and Nyachoti [26] reported that when pigs consumed more than 2 times of maintenance energy, the BEL of CP (g/kg DMI) remained constant in pigs weighing greater than 60 kg, which is concordance with the results in this study (Figure 2). This result may have occurred because the increased BEL of CP in response to increased DMI occupies a relatively small proportion. The influence of FI:MFI on BEL of CP (g/kg DMI) in pigs is clear [22,25] particularly when the FI:MFI is extremely low (e.g., FI:MFI = 1) and the previous equation by Park et al [11] adopted the FI:MFI as an independent variable for estimating BEL of CP. In the present study, however, data with extremely low FI:MFI were excluded from the dataset based on the Cook’s distance test, which may explain why FI:MFI was not included in the novel equations for estimating BEL of CP.

In the present study, the prediction equations for estimating BEL of CP (g/kg DMI) were developed using linear and exponential models with IBW as an independent variable. Both models had similar root mean squares of error and determination coefficients. These model calculations should precede the estimating BEL of AA as the linear model for the BEL of AA contain BEL of CP as an independent variable.

## CONCLUSION

The accuracy of equations for estimating BEL of CP in pigs has been improved in the present study. In the novel linear models for estimating BEL of AA of pigs, BEL of CP were used as an independent variable. The novel prediction equations for estimating the BEL of CP and AA can be used when calculating SID of CP and AA.

## Figures and Tables

**Table 1:** Variability of initial body weight of pigs and basal endogenous losses of crude protein and amino acids

Item	n	Mean	Minimum	Maximum	SD	CV
IBW (kg)	139	43.2	9.2	109.8	22.2	51.3
Feed intake (kg/d)	139	1.448	0.405	2.812	0.584	40.3
Metabolizable energy intake (kcal/d)	139	5,426	1,535	10,786	2,182	40.2
FI:MFI^1)^	139	2.73	1.91	3.00	0.27	10.1
BEL of CP and all AA (g/kg DMI)						
CP	117	17.1	6.1	29.1	5.2	30.2
BEL of indispensable AA						
Arg	139	0.589	0.043	1.290	0.243	41.2
His	139	0.190	0.060	0.550	0.072	37.7
Ile	139	0.322	0.130	0.690	0.106	32.9
Leu	139	0.519	0.220	1.070	0.157	30.2
Lys	139	0.424	0.050	0.880	0.160	37.8
Met	138	0.097	0.020	0.360	0.049	50.5
Phe	139	0.333	0.060	0.860	0.124	37.3
Thr	138	0.545	0.110	1.210	0.159	29.1
Trp	128	0.131	0.040	0.600	0.076	58.0
Val	138	0.470	0.100	0.910	0.148	31.4
BEL of dispensable AA						
Ala	137	0.592	0.070	1.160	0.184	31.1
Asp	135	0.785	0.320	1.840	0.223	28.4
Cys	132	0.202	0.026	0.700	0.088	43.3
Glu	134	0.973	0.200	2.320	0.313	32.2
Gly	134	1.536	0.340	3.950	0.593	38.6
Pro	115	4.542	0.290	14.20	2.954	65.0
Ser	135	0.565	0.210	5.150	0.442	78.2
Tyr	106	0.274	0.060	0.690	0.100	36.7

SD, standard deviation; CV, coefficient of variation; IBW, initial body weight; FI:MFI, feed intake-to-maintenance feed intake ratio; DMI, dry matter intake; BEL, basal endogenous losses; CP, crude protein; AA, amino acids.

1) When the value of FI:MFI in each experiment was greater than the value calculated using the NRC [12], the FI:MFI was used as calculated value by using the NRC [12].

**Table 2:** Correlation coefficients among initial body weight, feed intake-to-maintenance feed intake ratio, and basal endogenous losses of crude protein and amino acids^1)^

Item	IBW	FI:MFI	Basal endogenous losses									

			CP	Arg	His	Ile	Leu	Lys	Met	Phe	Thr	Trp
FI:MFI	0.40^***^											
CP	−0.34^***^	−0.20^*^										
Arg	−0.25^**^	−0.11	0.86^***^									
His	−0.39^***^	−0.31^***^	0.59^***^	0.55^***^								
Ile	−0.34^***^	−0.35^***^	0.55^***^	0.47^***^	0.68^***^							
Leu	−0.40^***^	−0.41^***^	0.59^***^	0.43^***^	0.59^***^	0.72^***^						
Lys	−0.37^***^	−0.26^**^	0.53^***^	0.50^***^	0.65^***^	0.71^***^	0.72^***^					
Met	−0.22^**^	−0.20^*^	0.25^**^	0.11	0.60^***^	0.48^***^	0.46^***^	0.41^***^				
Phe	−0.37^***^	−0.36^***^	0.47^***^	0.41^***^	0.65^***^	0.78^***^	0.72^***^	0.62^***^	0.35^***^			
Thr	−0.37^***^	−0.37^***^	0.66^***^	0.47^***^	0.55^***^	0.63^***^	0.81^***^	0.56^***^	0.41^***^	0.62^***^		
Trp	−0.17^*^	−0.09	0.32^***^	0.22^*^	0.68^***^	0.36^***^	0.15	0.30^***^	0.62^***^	0.26^**^	0.21^*^	
Val	−0.38^***^	−0.38^***^	0.67^***^	0.52^***^	0.52^***^	0.74^***^	0.84^***^	0.68^***^	0.43^***^	0.61^***^	0.79^***^	0.16

IBW, initial body weight; FI:MFI, feed intake-to-maintenance feed intake ratio; CP, crude protein.

1) The number of observations for IBW, FI:MFI, and BEL of Arg, His, Ile, Leu, Lys, and Phe was 139. The BEL of Met, Thr, and Val were from 138 data and that of Trp was from 128 data.

* p<0.05,

** p<0.01,

*** p<0.001.

**Table 3:** Prediction equations for estimating basal endogenous losses of crude protein based on feed intake-to-maintenance feed intake ratio and initial body weight of pigs (n = 117)^1)^

Model type	C_1_	C_2_	C_3_	RMSE	R^2^	p-value
Linear^2)^	20.36^***^	−0.077^***^	-	4.87	0.11	<0.001
	(0.97)	(0.020)	-			
	27.38^***^	-	−3.790^*^	5.07	0.04	0.031
	(4.7^3)^	-	(1.73^1)^			
	21.64^***^	−0.074^**^	−0.522	4.89	0.11	0.001
	(4.9^3)^	(0.024)	(1.977)			
Exponential^3)^	20.80^***^	−0.00475^***^	-	4.86	0.12	<0.001
	(1.14)	(0.00126)	-			
	31.26^***^	-	−0.223^*^	5.07	0.04	<0.001
	(8.17)	-	(0.097)			
	22.21^***^	−0.00454^**^	−0.027	4.88	0.12	<0.001
	(6.19)	(0.0015^3)^	(0.114)			

RMSE, root mean square error; BEL, basal endogenous losses; CP, crude protein; IBW, initial body weight.

1) Values in parentheses are standard error.

2) BEL of CP = C_1_+C_2_×IBW+C_3_×FI:MFI, where BEL of CP expressed as g/kg dry matter intake and IBW as kg.

3) BEL of CP = C_1_×e^(C2×IBW+C3×FI:MFI)^, where BEL of CP expressed as g/kg dry matter intake and IBW as kg.

* p<0.05,

** p<0.01,

*** p<0.001.

**Table 4:** Prediction equations for estimating basal endogenous losses of amino acids based on basal endogenous losses of crude protein

Item (g/kg DMI)	Regression coefficient		Statistical parameter		
	
	Intercept	Slope	RMSE	r^2^	p-value
BEL of indispensable AA					
Arg (n = 117)	−0.090	0.041	0.124	0.74	<0.001
SE	0.040	0.002			
p-value	0.026	<0.001			
His (n = 117)	0.060	0.008	0.055	0.35	<0.001
SE	0.018	0.001			
p-value	0.001	<0.001			
Ile (n = 117)	0.140	0.011	0.086	0.31	<0.001
SE	0.028	0.002			
p-value	<0.001	<0.001			
Leu (n = 117)	0.231	0.018	0.125	0.35	<0.001
SE	0.040	0.002			
p-value	<0.001	<0.001			
Lys (n = 117)	0.148	0.017	0.138	0.28	<0.001
SE	0.044	0.002			
p-value	0.001	<0.001			
Met (n = 117)	0.059	0.002	0.046	0.06	0.007
SE	0.015	0.001			
p-value	<0.001	0.007			
Phe (n = 117)	0.159	0.011	0.103	0.22	<0.001
SE	0.033	0.002			
p-value	<0.001	<0.001			
Thr (n = 117)	0.217	0.020	0.118	0.43	<0.001
SE	0.038	0.002			
p-value	<0.001	<0.001			
Trp (n = 110)	0.058	0.004	0.064	0.10	0.001
SE	0.021	0.001			
p-value	0.008	0.001			
Val (n = 117)	0.145	0.019	0.112	0.45	<0.001
SE	0.036	0.002			
p-value	<0.001	<0.001			
BEL of dispensable AA					
Ala (n = 116)	0.098	0.030	0.093	0.73	<0.001
SE	0.030	0.002			
p-value	0.002	<0.001			
Asp (n = 114)	0.286	0.030	0.153	0.52	<0.001
SE	0.050	0.003			
p-value	<0.001	<0.001			
Cys (n = 113)	0.086	0.007	0.076	0.19	<0.001
SE	0.025	0.001			
p-value	0.001	<0.001			
Glu (n = 113)	0.376	0.037	0.245	0.38	<0.001
SE	0.079	0.004			
p-value	<0.001	<0.001			
Gly (n = 113)	−0.181	0.103	0.267	0.80	<0.001
SE	0.088	0.005			
p-value	0.041	<0.001			
Pro (n = 98)	−3.003	0.461	1.876	0.63	<0.001
SE	0.639	0.036			
p-value	<0.001	<0.001			
Ser (n = 114)	0.231	0.021	0.466	0.05	0.016
SE	0.151	0.008			
p-value	0.128	0.016			
Tyr (n = 86)	0.211	0.021	0.425	0.06	<0.001
SE	0.121	0.007			
p-value	0.083	0.003			

BEL, basal endogenous losses; AA, amino acids; DMI, dry matter intake; RMSE, root means square of error; SE, standard error.
